# Ivabradine Ameliorates Cardiac Function in Heart Failure with Preserved and Reduced Ejection Fraction via Upregulation of miR-133a

**DOI:** 10.1155/2021/1257283

**Published:** 2021-09-29

**Authors:** Shuai Shao, Yue Zhang, Mengqi Gong, Qian Yang, Meng Yuan, Ming Yuan, Ya Suo, Xinghua Wang, Ying Li, Qiankun Bao, Guangping Li

**Affiliations:** Tianjin Key Laboratory of Ionic-Molecular Function of Cardiovascular Disease, Department of Cardiology, Tianjin Institute of Cardiology, The Second Hospital of Tianjin Medical University, Tianjin 300211, China

## Abstract

Heart failure (HF) is a clinical syndrome caused by impairment of ventricular filling, ejection of blood, or both and is categorized as HF with reduced ejection fraction (HFrEF) or HF with preserved ejection fraction (HFpEF) based on left ventricular function. Cardiac fibrosis contributes to left ventricular dysfunction and leads to the development of HF. Ivabradine, an If current selective specific inhibitor, has been shown to improve the prognosis of patients with HF. However, the effects of ivabradine on cardiac function and fibrosis in HFpEF and HFrEF and the underlying mechanism remain unclear. In the present study, we utilized mouse models to mimic HFpEF and HFrEF and evaluated the therapeutic effects of ivabradine. By treating mice with different doses (10 mg/kg/d and 20 mg/kg/d) of ivabradine for 4 or 8 weeks, we found that a high dose of ivabradine improved cardiac diastolic function in HFpEF mice and ameliorated cardiac diastolic and systolic function and ventricular tachycardia incidence in HFrEF mice. Moreover, ivabradine significantly reduced the activation of cardiac fibroblasts and myocardial fibrosis in mice. Mechanistically, microRNA-133a, which was upregulated by ivabradine, targeted connective tissue growth factor and collagen 1 in cardiac fibroblasts and might contribute to the protective role of ivabradine. Together, our work utilized mouse models to study HFpEF and HFrEF, demonstrated the protective role of ivabradine in HFpEF and HFrEF, and elucidated the potential underlying mechanism, which provides an effective strategy for related diseases.

## 1. Introduction

Heart failure (HF) is a difficult problem for cardiologists in clinical practice. It has the characteristics of high incidence, high readmission rates, and high mortality, which seriously affect the quality of life of patients [1]. Based on the left ventricular function, HF could be further categorized as HF with reduced ejection fraction (HFrEF, also known as systolic HF) and HF with preserved ejection fraction (HFpEF, also known as diastolic HF). This differentiation is necessary for the clinic because of different underlying etiologies, demographics, comorbidities, and responses to therapies [2]. HFpEF implies a myocardial process impairing filling due to an increase in ventricular stiffness or a decrease in ventricular relaxation [3]. In both HFpEF and HFrEF, the activation of cardiac fibroblasts and myofibroblast formation contribute to maladaptive remodeling and progressive cardiac functional decline [4]. Numerous profibrotic factors have been identified at the molecular level and regulate gene expression programs for cardiac fibroblast activation [5, 6]. Our previous study showed that intermittent hypoxia triggered cardiac fibrosis and aggravated angiotensin II- (Ang II-) induced cardiac dysfunction in mice via upregulation of TSP1 and activation of transforming growth factor *β* (TGF-*β*) signaling [7]. However, there is much less emphasis on cardiac fibroblast activation as a key driver in pathogenic development from HFpEF to HFrEF.

An I(f) channel is a member of the hyperpolarization-activated cyclic nucleotide-gated (HCN) channel family, and it is regulated by the autonomic nervous system [8, 9]. Ivabradine is a selective specific inhibitor of the If current in the sinoatrial node [10]. HF is associated with an autonomic imbalance, resulting in an increase in heart rate with reduced heart rate variability parameters [11]. Ivabradine significantly increases vagal modulation and shifts the sympatho-vagal balance toward vagal dominance [12]. In the SHIFT Holter substudy, ivabradine safely and significantly lowers the heart rate and improves heart rate variability in patients with systolic heart failure, without inducing significant bradycardia, ventricular arrhythmias, or supraventricular arrhythmias [11].The current guidelines indicate that the application of ivabradine in patients with HFrEF has significant benefits in reducing the readmission rate and the main composite endpoint of cardiovascular death [1, 13, 14]. Besides, selective HR reduction by ivabradine significantly improved left ventricular contractility and diastolic function in diabetic db/db mice which showed features of HFpEF [15]. However, the direct role of ivabradine on pressure overload-induced HFpEF and HFrEF and the underlying mechanism remain to be elucidated.

Emerging evidence demonstrates that microRNAs (miRNAs) play an important role in transcription and translation modification in myocardial hypertrophy and fibrosis-related gene expression [16, 17]. miR-133a is highly expressed in the myocardium and regulates cardiac hypertrophy and myocardial fibrosis [18–22]. Connective tissue growth factor (CTGF) and collagen 1 (Col 1) are targets of miR-133a, which mediates Ang II-induced cardiac fibrosis [20, 23]. However, the upstream regulation of miR-133a remains unknown.

In the present study, we constructed a transverse aortic constriction (TAC) mouse model to mimic the different stages of HF and determined the effects of ivabradine on cardiac function and myocardial fibrosis in both HFpEF and HFrEF mice. Furthermore, we demonstrated that miR-133a might mediate the protective role of ivabradine in fibroblast activation and proliferation.

## 2. Materials and Methods

### 2.1. Animals

All animal procedures were approved and conducted in accordance with the experimental animal administration committee of Tianjin Medical University. Male C57BL/6J mice (8 weeks old) were used. All mice were housed in a controlled environment (20 ± 2°C, 12 h/12 h light/dark cycle) and maintained on a standard chow diet with free access to water. The TAC technique was used to establish a pressure overload-induced heart failure model. Four weeks after TAC surgery, the mice were treated with low-dose (10 mg/kg/d) and high-dose (20 mg/kg/d) ivabradine or saline by gavage for another 4 weeks to mimic HFpEF, and the mice undergoing sham surgery were established at the same time. Nine weeks after TAC surgery, the mice were treated with low-dose (10 mg/kg/d) and high-dose (20 mg/kg/d) ivabradine or saline by gavage for another 8 weeks to mimic HFrEF, and the mice undergoing sham surgery were established at the same time.

### 2.2. Transverse Aortic Constriction (TAC) Surgical Preparation

The TAC surgery used in our study was based on previous protocols [24–27] with modifications. Briefly, the mice were anesthetized by intraperitoneal injection of tribromoethanol (1.5%, 10 mL/kg). After satisfactory anesthesia, the mice were fixed on the operating table (36–37°C), routinely treated with hair removal disinfection, and intubated and connected to a ventilator. Local thoracotomy was performed in the second intercostal space of the left chest. The aortic arch between the left common carotid artery and brachiocephalic artery was identified, and a 26-gauge needle was ligated using a novel ligator. After ligation, the needle was quickly removed. After the operation, the ventilator was disconnected, endotracheal intubation was removed, and the mice were kept in a warm cage until they woke up. The sham mice underwent the same procedure without ligation.

### 2.3. Echocardiography

Transthoracic m-mode echocardiography was performed on all mice using an ultrasound system (Visual Sonics Vevo 2100, SONICS, Canada) as previously reported [7]. Briefly, mice were anesthetized by inhalation of isoflurane and fixed in a supine position on a warm platform (37°C). The hair on the chest area was removed using depilatory cream, a small amount of coupling agent was applied to the chest area, and the probe was placed on the left chest. An average of 5 cardiac cycles of the left ventricular long axis, left ventricular short axis, and aortic arch was measured in order to calculate interventricular septal thickness at diastole (IVSTd), left ventricular posterior wall thickness at diastole (LVPWd), left ventricular internal diameter at end-diastole (LVIDd), left ventricular internal diameter at end-systole (LVIDs), isovolumic relaxation time (IVRT), E peak deceleration time (EDT), left ventricular ejection fraction (LVEF), and fractional shortening (FS).

### 2.4. Hemodynamic Assessment

Open-chest and retrograde catheterization through the left ventricular apex approach was performed using a Millar catheter with MPVS ULTRA, as previously reported [28]. Briefly, mice were anesthetized by intraperitoneal injection of tribromoethanol, and chest hair was removed after mice were fixed in a supine position on a warm platform (37°C). The chest was opened after intubation of the trachea and connected to the ventilator. The apex of the left ventricle of mice was stabbed using a 25-gauge needle, and a Millar catheter was inserted into the left ventricular cavity through the stab wound. Left ventricular end-diastolic pressure (LVEDP), maximal left ventricular pressure rise (+dp/dtmax), and maximal rate of pressure fall (-dp/dtmax) were measured (Lab Chart Pro 8.0).

### 2.5. Electrophysiological Study

Ventricular electrical conduction heterogeneity and ventricular tachycardia (VT) induction rates were detected in Langendorff-perfused hearts by electrical mapping systems, as previously reported [29–31]. Briefly, mice were anesthetized by intraperitoneal injection of tribromoethanol. The hearts of mice were quickly isolated and placed in Tyrode's solution at 4°C. The heart without surrounding tissue was connected to the Langendorff perfusion system, which was filled with Tyrode's solution (37 ± 0.5°C, pH 7.35 ± 0.05, perfusion rate 2–3 mL/min). Epicardial activating electrical mapping was recorded using a 6 × 6 microelectrode (multielectrode probe assay) on the epicardial surface of the LV and RV. Data were recorded using a multichannel system (EMS64-USB-1003, United Kingdom). Conduction velocity (CV), absolute inhomogeneity, and inhomogeneity index were calculated using EMapScope 4.0 software (MappingLab Ltd., UK).

Two pairs of spiral contact electrodes were fixed in the left ventricle (LV) and right ventricle (RV) to record the sinus heart circumference and determine the pacing threshold. The basic cycle lengths of basic stimuli were 200 ms. Eight basic stimuli were followed by a premature extra stimulus to measure the ventricular effective refractory period. VT induction was verified by burst pacing with cycle lengths of 200 ms for 3 s, which was performed five times at 30 s intervals. Once VT induction was unsuccessful, cycle lengths were decreased by 10 ms, until VT was successfully induced or ventricular rhythm was not 1 : 1 following the atrial rhythm.

### 2.6. Histology

Heart tissues were soaked in 10% neutral-buffered formalin for 24 h at room temperature, wrapped in paraffin, and cut into 5 *μ*m cross sections for staining. Morphological changes were observed via hematoxylin and eosin (HE) staining, and collagen deposition was observed with Masson (Sigma-Aldrich, MO, USA) and Sirius red (Solarbio Life Science, Beijing) staining. Images of the sections were captured using an Olympus inverted microscope (IX53, Tokyo). Fibrotic areas were calculated using ImageJ 1.52a.5.

For immunofluorescence staining, sections were stained with primary antibodies against CTGF (1 : 100, sc-365970, Santa Cruz) and *α*-SMA (1 : 100, ab7817, Abcam) overnight at 4°C and then stained with Alexa 488-conjugated goat anti-mouse antibodies as secondary antibodies. Nuclei were stained with DAPI. Fluorescence intensity was quantified using ImageJ 1.52a.5.

### 2.7. Isolation and Culture of Primary Ventricular Fibroblasts (PVFs)

PVFs were isolated from neonatal Sprague-Dawley (SD) rats. Briefly, the left ventricle was separated from the exposed heart of neonatal SD rats sacrificed by cervical dislocation and minced to 1 mm^3^ in cold phosphate-buffered saline (PBS). Minced tissue was subsequently digested with trypsin type II collagenase buffer until the tissue was completely dissipated. The collected cell suspensions were centrifuged for 5 min at 1000 rpm. The isolated cells were resuspended in DMEM supplemented with 10% fetal bovine serum (FBS) and 100 IU/mL penicillin-streptomycin. The resuspended cells were plated into dishes and incubated for 2 h. Supernatants were discarded, and dishes were replenished with fresh medium. PVFs were incubated in a humidified atmosphere at 37°C and 5% CO_2_. PVFs at passages 3 to 5 were used in subsequent experiments and incubated with FBS-free medium for 24 h before treatment. PVFs were stimulated with Ang II (1 *μ*M) for 24 h and treated with ivabradine (3 *μ*M or 10 *μ*M) for 48 h.

### 2.8. RT-qPCR

RNA from frozen LV tissue and cultured PVFs were extracted using the Eastep Super Total RNA Extraction Kit (LS1040, Promega, WI). cDNA was synthesized using the iScript cDNA Synthesis Kit (Bio-Rad, CA, USA). The qPCR primers used to evaluate the mRNA levels of target genes are described in Supplementary Table 1.

### 2.9. Western Blot Analysis

Total proteins were extracted from frozen LV tissue and cultured PVFs using RIPA lysis buffer. Proteins were quantified using a BCA Protein Assay Kit (Thermo Fisher Scientific, USA). All sample proteins loaded equally (20 *μ*g) were separated by SDS-PAGE and transferred to PVDF membranes. Subsequently, the PVDF membrane was blocked with TBST containing 5% BSA for 1 h and incubated at 4°C overnight with a primary antibody. Then, the membrane was washed with TBST and incubated with a horseradish peroxidase-conjugated secondary antibody for 1 h. The primary antibodies used were as follows: GAPDH (1 : 5000, 60004-1-Ig, Proteintech), CTGF (1 : 1000, sc-365970, Santa Cruz), *α*-SMA (1 : 1000, ab7817, Abcam), Col 1 (1 : 1000, ab34710, Abcam), Col 3 (1 : 1000, ab7778, Abcam), TGF-*β*1 (1 : 1000, ab215715, Abcam), TGFR-2 (1 : 1000, ab269279, Abcam), Smad2 (1 : 1000, ab33875, Abcam), Smad3 (1 : 1000, ab40854, Abcam), and phospho-Smad2/3 (1 : 1000, ab272332, Abcam). Immunoblots were quantified using a Tanon 5200 Multi Chemiluminescent Imaging System (Tanon Science & Technology Co., Ltd., Shanghai, China).

### 2.10. miR-133a Mimic and Inhibitor Transfection

PVFs were seeded on plates, cultured to 80% confluence, and subsequently transfected with the miR133a_3p inhibitor (5′-CAGCUGGUUGAAGGGGACCAAA-3′) or miR133a_3p mimics (5′-UUUGGUCCCCUUCAACCAGCUG-3′/5′-GCUGGUUGAAGGGGACCAAAUU-3′) (GenePharma, Shanghai) using Lipofectamine 3000 for 24 h.

### 2.11. Statistical Analysis

Sample sizes were designed with adequate power, according to the literature and our previous studies. Data are presented as the mean ± standard error of the mean (SEM). Statistical analysis was performed using GraphPad Prism 7 v7.04. For normally distributed data, unpaired Student's *t*-test was used for two-group analysis. One-way ANOVA with the Bonferroni multiple comparison posttest was used for multiple-group analysis, and two-way ANOVA with the Bonferroni multiple comparison posttest was used for analysis of experiments with two independent variables. For nonnormally distributed data, the Kruskal-Wallis test with the Dunn multiple comparison test was used for multiple-group analysis. The criterion for statistical significance was set at *p* < 0.05.

## 3. Results

### 3.1. HFpEF and HFrEF Were Induced in Mice by a TAC Surgery

**Figure 1:**
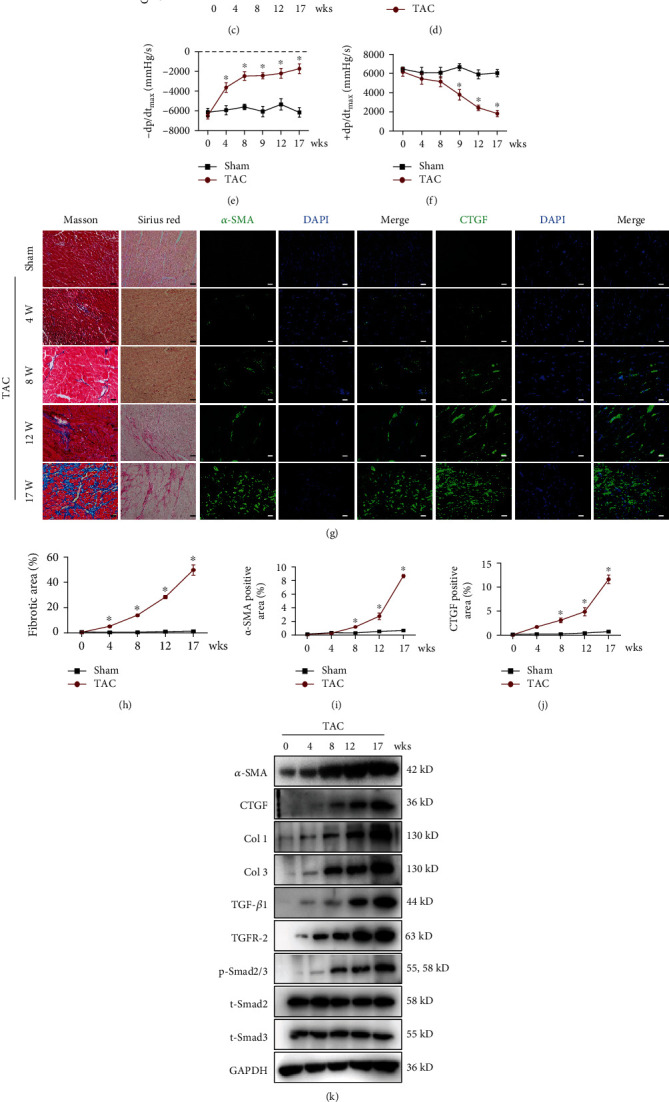
The construction of a transverse aortic constriction (TAC) model to mimic heart failure with preserved ejection fraction (HFpEF) and heart failure with reduced ejection fraction (HFrEF).C57BL/6 mice underwent TAC surgery for the indicated times. (a) Ratio of heart weight (HW) to tibial length (TL) in each group. (b) Representative images of HE staining of the left ventricle. Scale bar, 20 *μ*m. (c) Quantification of the cross-sectional area of the left ventricular cardiomyocytes in (b). (d–f) Left ventricular end-diastolic pressure (LVEDP), decreased maximal rate of pressure fall (-dp/dt_max_), and maximal left ventricular pressure rise (+dp/dt_max_) of mice were measured by hemodynamic assessment. Data are mean ± SEM (*n* = 9 mice per group). ^∗^Compared with the 0 week, *p* < 0.05, Kruskal-Wallis test with the Dunn multiple comparison test. (g) Representative images of Masson and Sirius red staining of the left ventricle (scale bar, 50 *μ*m) and representative confocal microscopy images of immunofluorescence staining for *α*-SMA, connective tissue growth factor (CTGF), and DAPI of the left ventricle (scale bar, 20 *μ*m). (h–j) Quantification of the fibrotic area and quantification of *α*-SMA and CTGF fluorescence intensity in (g). (k) Protein levels of *α*-SMA, CTGF, collagen 1 (Col 1), Col 3, transforming growth factor *β*1 (TGF-*β*1), TGF-*β* receptor 2 (TGFR-2), phosphorylated Smad2/3 (p-Smad2/3), total Smad2 (t-Smad2), and t-Smad3 in the left ventricle of mice detected by western blot analysis. (l) Quantification of *α*-SMA, CTGF, Col 1, Col 3, TGF-*β*1, TGFR-2, p-Smad2/3, t-Smad2, and t-Smad3 in (k). Data are mean ± SEM (*n* = 9 mice per group). ^∗^Compared with the 0 week, *p* < 0.05, one-way ANOVA with the Bonferroni posttest.

The animal model of TAC, which mimics human aortic stenosis, is a well-defined model that induces cardiac hypertrophy and HF caused by pressure overload, leading to decreased ventricular function in mice [32, 33]. To investigate the effect of ivabradine on HFpEF and HFrEF, we initially constructed a moderate TAC model in mice as previously reported [27]. We found upregulation of interventricular septal thickness at diastole (IVSTd), left ventricular posterior wall thickness at diastole (LVPWd), diastolic blood pressure (DBP), and systolic blood pressure (SBP) in mice from 4 to 17 weeks after TAC surgery compared to sham-operated mice (Tables 1 and 2). Consistently, the ratio of heart weight to tibial length and cross-sectional areas of ventricular cardiomyocytes were also significantly increased from 4 to 17 weeks in mice after TAC compared to sham-operated mice (Figures 1(a)–1(c)). Next, a hemodynamic assessment was performed to analyze the cardiac function of mice after TAC surgery. The cardiac diastolic function was decreased from 4 to 17 weeks, as evidenced by increased left ventricular end-diastolic pressure (LVEDP) and decreased maximal rate of pressure fall (-dp/dt_max_) (Figures 1(d) and 1(e)). Simultaneously, isovolumic relaxation time (IVRT) and E peak deceleration time (EDT) increased from 4 weeks in mice after TAC surgery (Table 2). However, the maximal left ventricular pressure rise (+dp/dt_max_) was significantly decreased from the ninth week (Figure 1(f)), indicating that the cardiac systolic function of mice was impaired from the ninth week after TAC surgery. Moreover, left ventricular ejection fraction (LVEF) and fractional shortening (FS) of mice were also significantly downregulated from the ninth week (Table 2). Collagen deposition in the heart significantly increased from 4 weeks after TAC surgery (Figures 1(g) and 1(h)). Meanwhile, immunofluorescence staining showed that the expression of *α*-SMA and CTGF was significantly upregulated from the eighth week (Figures 1(g), 1(i), and 1(j)), indicating the activation of fibroblasts. In support of this, we found that the protein levels of *α*-SMA, CTGF, Col 1, Col 3, TGF-*β*1, and TGF-*β* receptor 2 (TGFR-2) and phosphorylation of Smad2/3 were markedly upregulated in a time-dependent manner in the left ventricle of mice after TAC (Figures 1(k) and 1(l)). Together, these results suggested that the moderate TAC mouse model showed typical features of both HFpEF (4-8 weeks after TAC) and HFrEF (9-17 weeks after TAC).

### 3.2. Ivabradine Attenuated the Cardiac Diastolic Dysfunction and Cardiac Fibrosis in HFpEF

**Figure 2:**
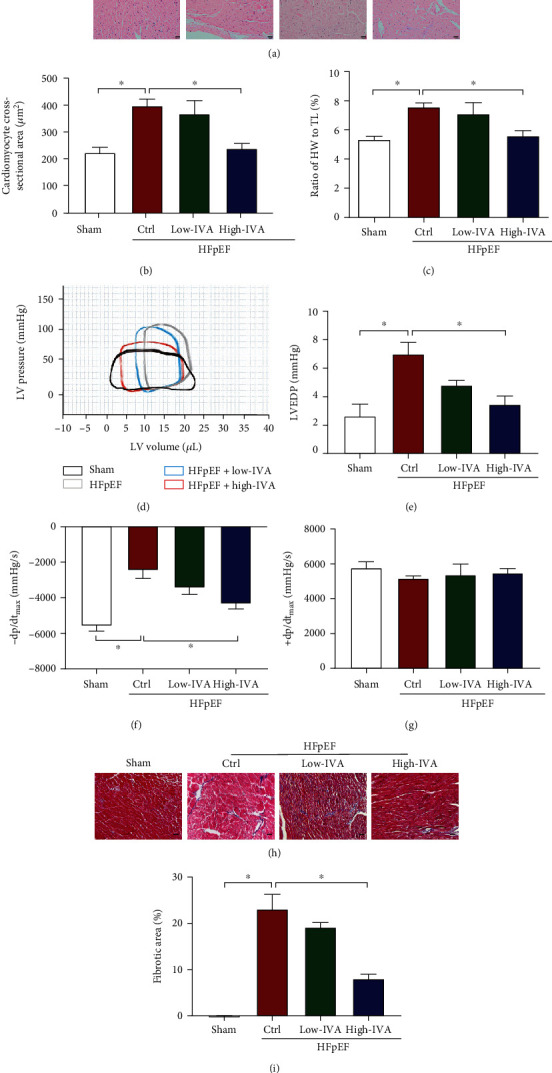
Ivabradine (IVA) attenuated cardiac diastolic dysfunction and cardiac fibrosis in HFpEF mice. Four weeks after TAC surgery, C57BL/6 mice were treated with low-dose (10 mg/kg/d) or high-dose (20 mg/kg/d) IVA for another 4 weeks. (a) Representative images of HE staining of the left ventricle in each group. Scale bar, 20 *μ*m. (b) Quantification of cross-sectional areas of left ventricular cardiomyocytes in (a). (c) Ratio of HW to TL in each group. (d) Representative pressure-volume loops in each group. (e–g) LVEDP, -dp/dt_max_, and +dp/dt_max_ of mice were measured by hemodynamic assessment in each group. (h) Representative images of Masson staining of the left ventricle. Scale bar, 50 *μ*m. (i) Quantification of the fibrotic area in (h). Data are presented as mean ± SEM (*n* = 9 mice per group); ^∗^*p* < 0.05, one-way ANOVA with the Bonferroni posttest.

To investigate the effect of ivabradine on HFpEF, we treated mice at 4-weeks post-TAC with low-dose (10 mg/kg/d) [34, 35] or high-dose (20 mg/kg/d) [34, 35] ivabradine for another 4 weeks. We found that high-dose but not low-dose ivabradine significantly inhibited pressure overload-induced cardiac hypertrophy by reducing the cross-sectional areas of ventricular cardiomyocytes and the ratio of heart weight to tibial length (Figures 2(a)–2(c)). In addition, high-dose but not low-dose ivabradine significantly decreased LVEDP and increased -dp/dt_max_ in mice (Figures 2(d)–2(f)). Consistently, the echocardiographic assessment revealed that high-dose ivabradine decreased IVSTd, LVPWd, IVRT, and EDT (Table 3). However, either low-dose or high-dose ivabradine had little effect on cardiac systolic function in hemodynamic and echocardiographic assessments (Figures 2(d) and 2(g) and Table 3). Cardiac fibrosis induced by TAC was also reduced by treatment with a high dose of ivabradine from 23.08% to 8.03% (Figures 2(h) and 2(i)). Hence, our data demonstrated that ivabradine could ameliorate cardiac diastolic dysfunction and cardiac fibrosis in the HFpEF stage.

### 3.3. Ivabradine Attenuated the Cardiac Dysfunction and Electrical Remodeling in HFrEF

**Figure 3:**
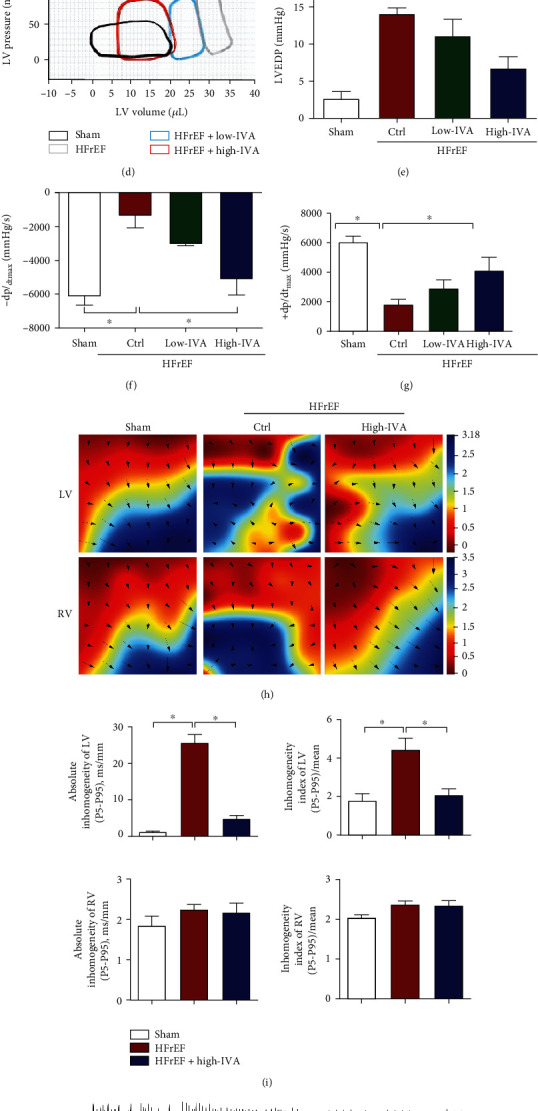
IVA ameliorated cardiac diastolic and systolic dysfunction in HFrEF mice. Nine weeks after TAC surgery, C57BL/6 mice were treated with low-dose (10 mg/kg/d) or high-dose (20 mg/kg/d) IVA for 8 weeks. (a) Representative images of HE staining of the left ventricle in each group. Scale bar, 20 *μ*m. (b) Quantification of cross-sectional areas of left ventricular cardiomyocytes in (a). (c) Ratio of HW to TL in each group. (d) Representative pressure-volume loops in each group. (e–g) LVEDP, -dp/dt_max_, and +dp/dt_max_ of mice were measured by hemodynamic assessment in each group. (h) Representative images of the conduction velocity and conduction direction of the left and right ventricles recorded by electrical mapping systems. (i) Absolute inhomogeneity and inhomogeneity index of the left ventricle (LV) and right ventricle (RV) in each group. (j) Representative images of the LV and RV tachycardia (VT) in HFrEF mice. (k) Rate of VT induction in each group. Data are presented as mean ± SEM (*n* = 9 mice per group); ^∗^*p* < 0.05, one-way ANOVA with the Bonferroni posttest.

Next, we investigated the effect of ivabradine on HFrEF. We treated the mice at 9 weeks post-TAC with low-dose (10 mg/kg/d) or high-dose (20 mg/kg/d) ivabradine for 8 weeks. High-dose ivabradine significantly inhibited cardiac hypertrophy by reducing the cross-sectional areas of ventricular cardiomyocytes and the ratio of heart weight to tibial length (Figures 3(a)–3(c)). High-dose ivabradine improved pressure overload-induced cardiac diastolic dysfunction with decreasing LVEDP and increasing -dp/dt_max_ (Figures 3(d)–3(f)). Consistently, high-dose ivabradine decreased IVRT and EDT under pressure overload in the echocardiographic assessment (Table 4). High-dose ivabradine improved pressure overload-impaired cardiac systolic function with increasing +dp/dt_max_, LVEF, and FS (Figures 3(d) and 3(g) and Table 4). Furthermore, we recorded electrical conduction mapping of the left ventricle (LV) and right ventricle (RV) from Langendorff-perfused hearts. LV conduction mode disorder and conduction heterogeneity were increased in HFrEF mice, with increasing absolute inhomogeneity (P5-P95, ms/mm) and inhomogeneity index ((P5-P95)/mean), which were largely reversed by high-dose ivabradine (Figures 3(h) and 3(i)). However, conduction mode disorder and conduction heterogeneity were not observed in the RV (Figures 3(h) and 3(i)). Next, the induction rate of ventricular tachycardia (VT) was significantly increased in HFrEF, and high-dose ivabradine decreased the induction rate (Figures 3(j) and 3(k)). Together, these results demonstrate that ivabradine could improve both cardiac diastolic dysfunction and cardiac systolic dysfunction induced by pressure overload.

### 3.4. Ivabradine Inhibited Cardiac Fibroblast Activation and Ameliorated Cardiac Fibrosis in HFrEF

**Figure 4:**
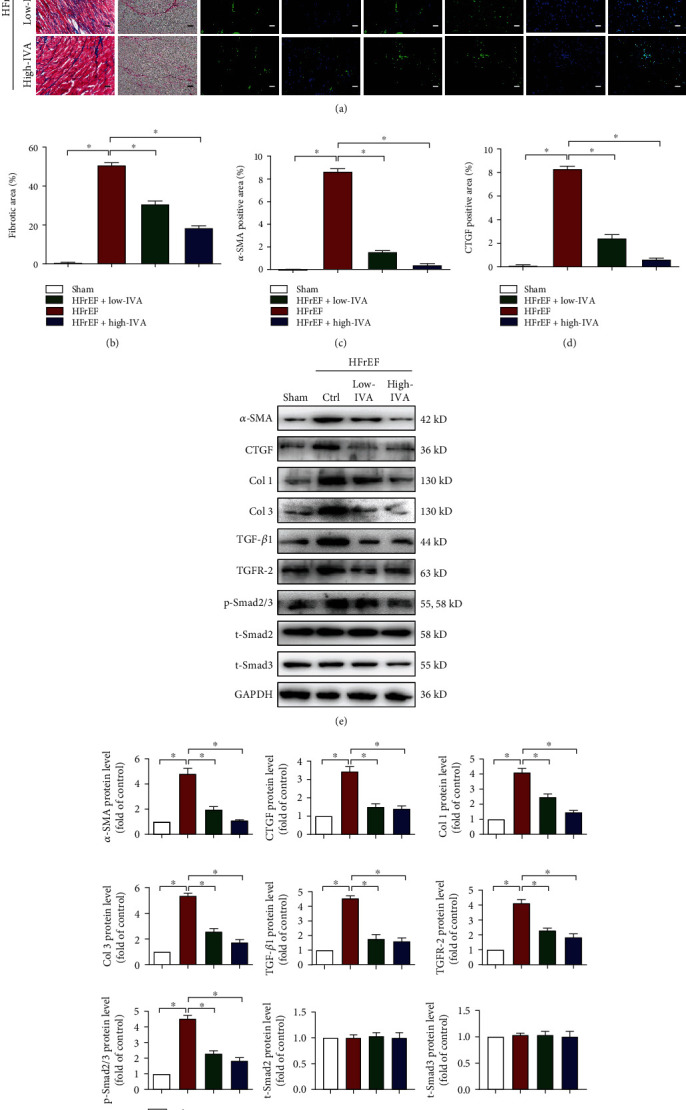
IVA decreased cardiac fibrosis in HFrEF mice. Nine weeks after TAC surgery, C57BL/6 mice were treated with low-dose (10 mg/kg/d) or high-dose (20 mg/kg/d) IVA for 8 weeks. (a) Representative images of Masson and Sirius red staining of the left ventricle (scale bar, 50 *μ*m) and representative confocal microscopy images of immunofluorescence staining for *α*-SMA, CTGF, and DAPI (scale bar, 20 *μ*m). (b–d) Quantification of the fibrotic area and quantification of *α*-SMA and CTGF fluorescence intensity in (a). (e) Protein levels of *α*-SMA, CTGF, Col 1, Col 3, TGF-*β*1, TGFR-2, p-Smad2/3, t-Smad2, and t-Smad3 in the left ventricle of mice detected by western blot analysis. (f) Quantification of *α*-SMA, CTGF, Col 1, Col 3, TGF-*β*1, TGFR-2, p-smad2/3, t-Smad2, and t-Smad3 in (e). Data are presented as mean ± SEM (*n* = 9 mice per group); ^∗^*p* < 0.05, one-way ANOVA with the Bonferroni posttest.

Next, we examined the effect of ivabradine on fibroblast activation and cardiac fibrosis in HFrEF mice. Masson and Sirius red staining showed that both low-dose and high-dose ivabradine ameliorated cardiac fibrosis under pressure overload (Figures 4(a) and 4(b)). Meanwhile, immunofluorescence staining showed that both low-dose and high-dose ivabradine ameliorated cardiac fibroblast activation by reducing *α*-SMA and CTGF signals in the left ventricle of HFrEF mice (Figures 4(a), 4(c), and 4(d)). Similarly, both low-dose and high-dose ivabradine reduced the expression of *α*-SMA, CTGF, Col 1, Col 3, TGF-*β*1, and TGFR-2 and phosphorylation of Smad2/3 in HFrEF mice (Figures 4(e) and 4(f)). Together, these results indicate that ivabradine inhibits cardiac fibroblast activation and ameliorates cardiac fibrosis.

### 3.5. Ivabradine Inhibited Cardiac Fibroblast Proliferation and Activation

**Figure 5:**
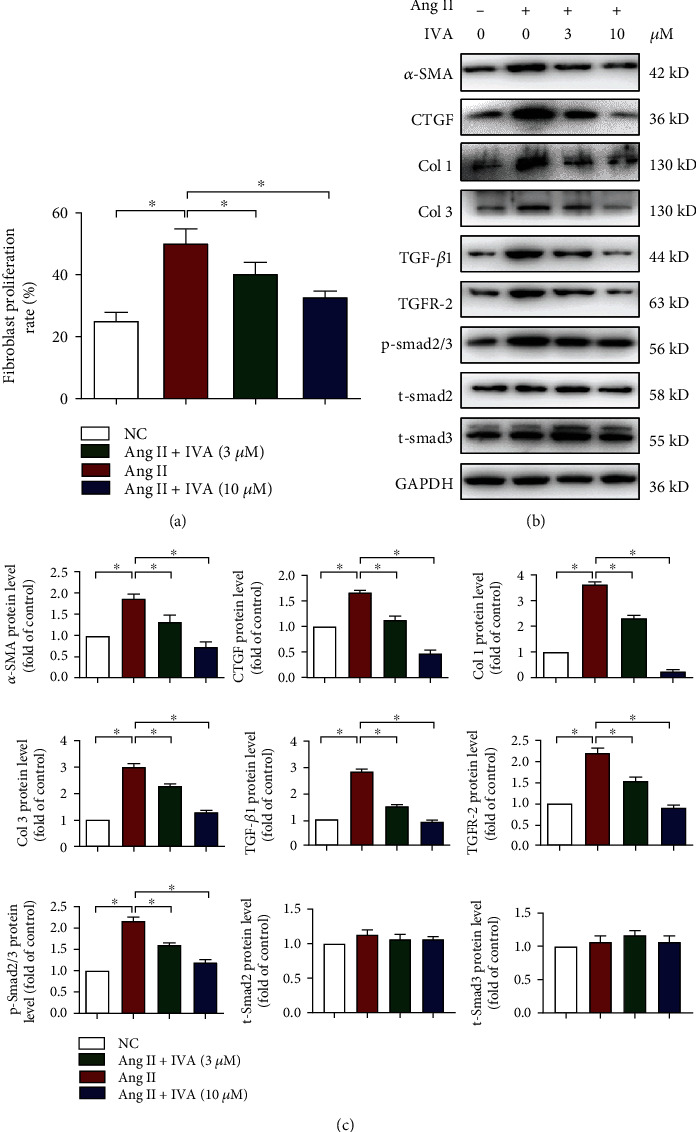
IVA inhibited cardiac fibroblast proliferation and activation. Primary ventricular fibroblasts (PVFs) were pretreated with angiotensin II (Ang II) for 24 h and treated with the indicated dose of IVA for 48 h. (a) The proliferation rate of PVFs in each group was detected by the CCK-8 assay. (b) Protein levels of *α*-SMA, CTGF, Col 1, Col 3, TGF-*β*1, TGFR-2, p-Smad2/3, t-Smad2, and t-Smad3 in the left ventricle of mice detected by western blot analysis. (c) Quantification of *α*-SMA, CTGF, Col 1, Col 3, TGF-*β*1, TGFR-2, p-smad2/3, t-Smad2, and t-Smad3 in (b). Data are presented as mean ± SEM (*n* = 5 independent experiments); ^∗^*p* < 0.05, one-way ANOVA with the Bonferroni posttest.

Given the remarkable inhibitory effect of ivabradine on fibrosis in both HFpEF and HFrEF mice, we next determined whether ivabradine exerted its effects on fibroblast activation. First, primary ventricular fibroblasts (PVFs) were isolated from neonatal Sprague-Dawley rats. PVF proliferation was detected using the CCK-8 assay. We found that both 3 and 10 *μ*M ivabradine significantly decreased Ang II-induced PVF proliferation (Figure 5(a)). Furthermore, ivabradine decreased Ang II-induced protein levels of *α*-SMA, CTGF, Col 1, Col 3, TGF-*β*1, and TGFR-2 and phosphorylation of Smad2/3 in PVFs (Figures 5(b) and 5(c)).

### 3.6. miR-133a Blunted PVF Proliferation and Activation

**Figure 6:**
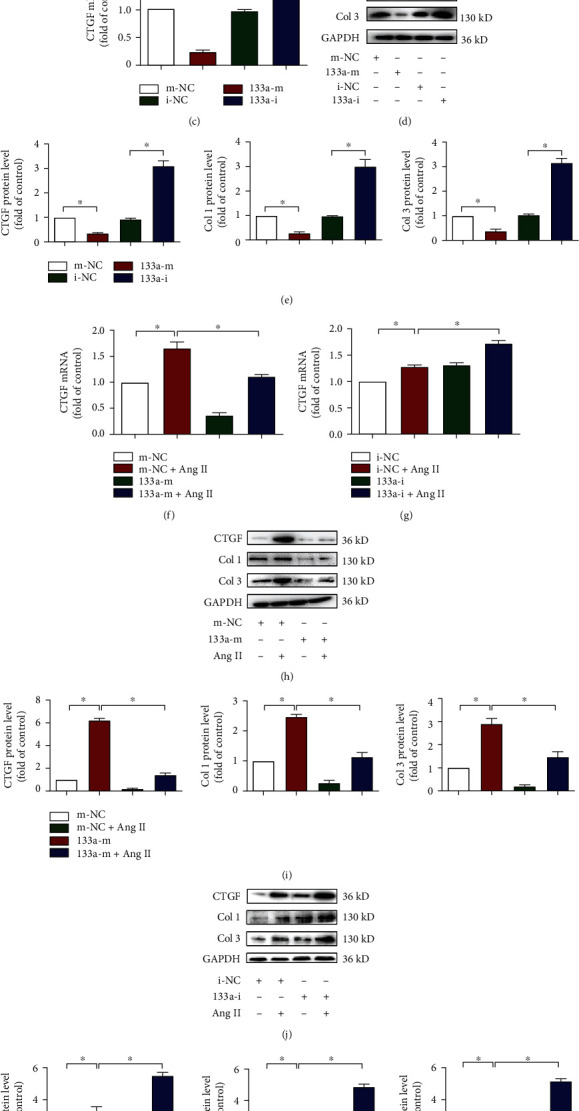
miR-133a blunted cardiac fibroblast proliferation and activation. (a) PVFs were treated with or without Ang II for 24 h. miR-133a level in PVFs was quantified by RT-PCR. Data are mean ± SEM (*n* = 5 independent experiments); ^∗^*p* < 0.05, unpaired 2-tail *t*-test. (b–e) PVFs were transfected with miR-133a mimics (133a-m) or negative control (m-NC) and inhibitors (133a-i) or negative control (i-NC) for 24 h. (b) The proliferation rate of PVFs in each group was detected by the CCK-8 assay. (c) CTGF mRNA levels in each group were quantified by RT-PCR. (d) The protein levels of CTGF, Col 1, and Col 3 were detected by western blot analysis. (e) Quantification of CTGF, Col 1, and Col 3 in (d). Data are mean ± SEM (*n* = 5 independent experiments); ^∗^*p* < 0.05, one-way ANOVA with the Bonferroni posttest. (f–k) PVFs were transfected with 133a-m or 133a-i for 24 h and treated with Ang II for 24 h. (f–g) CTGF mRNA levels in each group were quantified by RT-PCR. (h, j) The protein levels of CTGF, Col 1, and Col 3 were detected by western blot analysis. (i, k) Quantification of CTGF, Col 1, and Col 3 in (h) and (j). Data are mean ± SEM (*n* = 5 independent experiments); ^∗^*p* < 0.05, two-way ANOVA with the Bonferroni posttest.

miRNAs control structural changes in the extracellular matrix of the myocardium [17, 36]. miR-133a is decreased in pathological left ventricular hypertrophy [23], and miR-133a limits the production of CTGF and COL1A1 by directly targeting their 3′-UTR regions [20, 23, 37]. We next investigated whether miR-133a mediates the protective effects of ivabradine. We found that the expression of miR-133a was significantly decreased after Ang II exposure in PVFs (Figure 6(a)). Next, we overexpressed and inhibited miR-133a in PVFs and found that the miR-133a mimic decreased PVF proliferation, but the miR-133a inhibitor increased PVF proliferation (Figure 6(b)). Furthermore, overexpression of miR-133a decreased CTGF expression, while inhibition of miR-133a increased CTGF expression at both the mRNA and protein levels (Figures 6(c)–6(e)). Meanwhile, the protein levels of Col 1 and Col 3 showed a similar trend to that of CTGF (Figures 6(d) and 6(e)). In addition, miR-133a overexpression dramatically declined, while miR-133a inhibition significantly elevated the mRNA and protein levels of CTGF, Col 1, and Col 3 induced by Ang II (Figures 6(f)–6(k)). These results show that miR-133a negatively regulates the expression of CTGF and inhibits the proliferation and activation of PVFs.

### 3.7. Ivabradine Inhibited PVF Activation via Upregulation of miR-133a Expression

**Figure 7:**
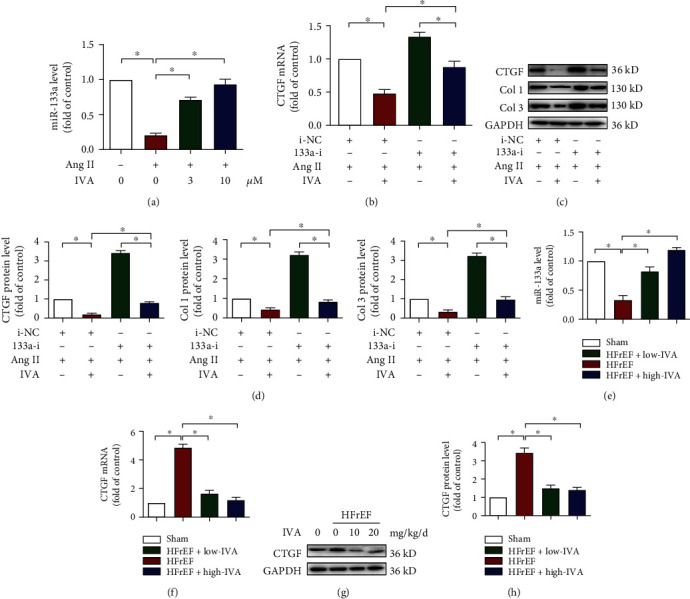
IVA inhibited cardiac fibroblast activation via upregulating miR-133a *in vitro* and *in vivo*. (a) PVFs were pretreated with Ang II for 24 h and administered the indicated dose of IVA for 48 h. miR-133a level was quantified by RT-PCR. Data are mean ± SEM (*n* = 5 independent experiments); ^∗^*p* < 0.05, one-way ANOVA with the Bonferroni posttest. (b–d) PVFs transfected with 133a-i for 24 h were pretreated with Ang II for 24 h and treated with or without IVA for 48 h. (b) CTGF mRNA was quantified by RT-PCR. (c) The protein levels of CTGF, Col 1, and Col 3 were detected by western blot analysis. (d) Quantification of CTGF, Col 1, and Col 3 in (c). Data are mean ± SEM (*n* = 5 independent experiments); ^∗^*p* < 0.05, two-way ANOVA with the Bonferroni posttest. (e–h) Nine weeks after TAC surgery, C57BL/6 mice were treated with low-dose (10 mg/kg/d) or high-dose (20 mg/kg/d) IVA for 8 weeks. (e) miR-133a level in the left ventricle was quantified by RT-PCR. (f) CTGF level in the left ventricle was quantified by RT-PCR. (g) The protein level of CTGF in the left ventricle was detected by western blot analysis. (h) Quantification of CTGF in (g). Data are mean ± SEM (*n* = 9 mice per group); ^∗^*p* < 0.05, one-way ANOVA with the Bonferroni posttest.

We next explored the effect of ivabradine on miR-133a in the activation of PVFs. We found that both 3 and 10 *μ*M ivabradine elevated the expression of miR-133a, which was impaired by Ang II (Figure 7(a)). The downregulation of both mRNA and protein levels of CTGF due to ivabradine was partially reversed by the miR-133 inhibitor (Figures 7(b)–7(d)). Simultaneously, Col 1 and Col 3 protein expression showed a trend similar to that of CTGF (Figures 7(c) and 7(d)). Consistently, both low-dose and high-dose ivabradine increased the expression of miR-133a (Figure 7(e)) and decreased the mRNA and protein levels of CTGF in the left ventricle of HFrEF mice (Figures 7(f)–7(h)). Taken together, our results indicate that the protective effects of ivabradine might be mediated, at least partially, by miR-133a.

## 4. Discussion

Epidemiological studies have consistently demonstrated an increased risk of all-cause and cardiovascular mortality in HFpEF patients compared to HF-free individuals [3, 38]. In the present study, we constructed a moderate TAC model to mimic the pathogenic process from HFpEF to HFrEF. Furthermore, we found that high-dose but not low-dose ivabradine ameliorated pressure overload-induced left ventricular diastolic dysfunction in HFpEF mice. In addition, ivabradine treatment in HFrEF mice for 8 weeks attenuated pressure overload-induced cardiac diastolic and systolic dysfunction and electrical remodeling. Moreover, ivabradine ameliorated cardiac fibrosis by inhibiting cardiac fibroblast activation through upregulating miR-133a in cardiac fibroblasts.

HFpEF is a complex clinical syndrome characterized by impairment of ventricular filling and has no effective treatment strategy [3, 39, 40]. Increased diastolic left ventricular stiffness, caused by excessive extracellular matrix accumulation in the myocardial interstitium, is a hallmark of HFpEF [41]. In previous studies, TAC surgery, deoxycorticosterone acetate pellet implantation, and combination of high-fat diet and inhibition of constitutive nitric oxide synthase were conducted to induce an HFpEF-like state [41–43]. In our study, we used a pressure-overloaded TAC model for the different stages of HF in mice. The moderate TAC initially leads to compensated hypertrophy of the left ventricle and HFpEF, which manifests as diastolic dysfunction. Over time, the response to chronic pressure overload becomes decompensation, resulting in ventricular dilatation and HFrEF, which manifests as systolic dysfunction.

Cardiac fibrosis is caused by an imbalance between the production and degradation of ECM and the stiffness of the left ventricle, thereby impairing systolic and diastolic function [44]. The proliferation and activation of resident cardiac fibroblasts, which differentiate into myofibroblasts in response to injury or stress, are key contributors to cardiac fibrosis [4]. Although myofibroblast formation is a physiological response to acute injuries, such as myocardial infarction and myofibroblast persistence, as occurs in HF, it contributes to maladaptive remodeling and progressive functional decline [4]. Cardiac fibroblasts are activated and differentiate into myofibroblasts, specifically showing *α*-SMA expression, leading to collagen deposition [45]. Collagen deposition triggers systolic and diastolic dysfunction and reduces left ventricular compliance [45]. In our study, ivabradine significantly inhibited Ang II-induced PVF proliferation and activation, which decreased Ang II-induced expression of the activated cardiac fibroblast marker *α*-SMA. Meanwhile, ivabradine decreased Ang II-induced profibrotic factors, CTGF and TGF-*β*1 expression, and downstream Smad2/3 signaling activation, further decreasing the expression of ECM structural proteins Col 1 and Col 3.

miRNAs play important roles in myocardial hypertrophy and fibrosis [46]. Among them, miR-133a repairs heart function in the HF model and promotes the repair and regeneration of cardiomyocytes [47]. The 3′-UTR of TGFBR1, CTGF, or COL1A1 contains a miR-133a binding site that is conserved among different species [20, 23, 37]. Here, we found that miR-133a overexpression was downregulated, while the miR-133a inhibitor further promoted Ang II-induced expression of CTGF, Col 1, and Col 3 in PVFs. Furthermore, ivabradine upregulated the expression of miR-133a under Ang II exposure and inhibited Ang II-induced PVF activation, suggesting that ivabradine inhibits cardiac fibroblast activation and ameliorates myocardial fibrosis by upregulating miR-133a expression. Besides heart rate reduction, ivabradine was also shown to exert effects within the vasculature in terms of inflammation and oxidative stress reduction [48, 49]. For example, ivabradine could diminish ROS generation in endothelial cells by provoking mTORC2/Akt phosphorylation [50]. In the present study, we found that ivabradine ameliorated cardiac fibrosis, at least partially, via upregulating miR-133a in cardiac fibroblasts, indicating that miR-133a might serve as a novel target of ivabradine in fibroblasts. However, as a limitation of the present study, the mechanism of upregulation of miR-133a induced by ivabradine is still unknown. Further studies to dissect the role of the I(f) channel in the process are warranted.

Cardiac fibrosis damages the mechanical-electrical coupling system, evokes trigger mechanisms, and results in arrhythmia and sudden death [51–53]. Our results revealed that the excitatory conduction of the ventricular tissue was abnormally disordered, and the normal wave-shaped conduction mode was disrupted, leading to a significant increase in the induction rate of ventricular tachycardia (VT) in HFrEF mice. Ivabradine induces heart rate reduction, which prolongs the left ventricular diastolic filling time [15]. In our study, both low-dose and high-dose ivabradine reduced the heart rate in mice with both HFpEF and HFrEF (Tables 3 and 4). Furthermore, ivabradine decreased left ventricular conduction heterogeneity and reduced the incidence of VT in mice with HFrEF. Our results indicated that the protective role of ivabradine on electrophysiological cardiac remodeling might be attributed to the amelioration of cardiac fibroblast activation and myocardial fibrosis via upregulation of miR-133a and downregulation of target genes CTGF and Col 1 of miR-133a.

## 5. Conclusions

In summary, our study demonstrated that ivabradine ameliorated pressure overload-induced myocardial fibrosis and cardiac dysfunction by upregulating miR-133a, indicating a potential therapeutic strategy for managing HFpEF and HFrEF.

## Figures and Tables

**Table 1:** Heart rate and blood pressure in mice after TAC surgery.

	0 week		4 weeks		8 weeks		12 weeks		17 weeks	
	Sham	TAC	Sham	TAC	Sham	TAC	Sham	TAC	Sham	TAC
HR (beats per min)	383.82 ± 40.08	384.95 ± 44.57	383.82 ± 40.08	406.74 ± 56.12	391.86 ± 53.11	478.31 ± 34.21^#^^∗^	392.38 ± 28.00	524.76 ± 32.63^#^^∗^	393.53 ± 26.27	559.27 ± 52.93^#^^∗^
DBP (mmHg)	78.18 ± 4.84	75.45 ± 9.76	74.92 ± 6.79	86.65 ± 4.47^#^^∗^	78.72 ± 11.34	92.98 ± 4.46^#^^∗^	75.84 ± 10.12	95.22 ± 9.30^#^^∗^	76.93 ± 7.45	99.22 ± 6.30^#^^∗^
SBP (mmHg)	120.95 ± 10.62	126.78 ± 9.24	127.44 ± 8.43	139.79 ± 6.53^#^^∗^	128.15 ± 7.59	151.70 ± 8.32^#^^∗^	127.61 ± 7.42	156.38 ± 8.55^#^^∗^	130.22 ± 7.31	163.63 ± 2.72^#^^∗^

Values are expressed as mean ± SEM (*n* = 9 mice per group). ^#^Compared with the 0 week, *p* < 0.05. ^∗^Compared with the sham group, *p* < 0.05, Kruskal-Wallis test with the Dunn multiple comparison test. HR: heart rate; DBP: diastolic blood pressure; SBP: systolic blood pressure.

**Table 2:** Echocardiography parameters in mice after TAC surgery.

	0 week		4 weeks		8 weeks		9 weeks		12 weeks		17 weeks	
	Sham	TAC	Sham	TAC	Sham	TAC	Sham	TAC	Sham	TAC	Sham	TAC
IVSTd (mm)	0.65 ± 0.04	0.66 ± 0.28	0.64 ± 0.03	0.76 ± 0.09^#^^∗^	0.65 ± 0.04	0.83 ± 0.06^#^^∗^	0.67 ± 0.03	0.87 ± 0.06^#^^∗^	0.65 ± 0.04	0.92 ± 0.04^#^^∗^	0.66 ± 0.04	0.95 ± 0.06^#^^∗^
LVPWd (mm)	0.66 ± 0.04	0.66 ± 0.03	0.67 ± 0.02	0.78 ± 0.06^#^^∗^	0.66 ± 0.03	0.82 ± 0.06^#^^∗^	0.67 ± 0.33	0.94 ± 0.08^#^^∗^	0.63 ± 0.01	0.97 ± 0.13^#^^∗^	0.65 ± 0.04	0.97 ± 0.09^#^^∗^
LVIDd (mm)	3.64 ± 0.13	3.62 ± 0.18	3.67 ± 0.14	3.58 ± 0.21	3.58 ± 0.11	3.85 ± 0.21	3.70 ± 0.17	3.96 ± 0.14^#^^∗^	3.71 ± 0.15	4.04 ± 0.25^#^^∗^	3.59 ± 0.21	4.22 ± 0.36^#^^∗^
LVIDs (mm)	2.12 ± 0.16	2.31 ± 0.23	2.21 ± 0.23	1.79 ± 0.25^#^^∗^	2.34 ± 0.32	2.42 ± 0.31	2.24 ± 0.21	3.30 ± 0.22^#^^∗^	2.43 ± 0.22	3.29 ± 0.17^#^^∗^	2.22 ± 0.24	3.44 ± 0.19^#^^∗^
IVRT (ms)	27.21 ± 5.02	26.30 ± 3.90	24.58 ± 3.74	38.28 ± 4.43^#^^∗^	28.02 ± 4.94	42.05 ± 8.32^#^^∗^	28.03 ± 3.54	43.82 ± 8.55^#^^∗^	28.89 ± 1.79	48.87 ± 7.86^#^^∗^	30.37 ± 4.12	58.89 ± 4.19^#^^∗^
EDT (ms)	32.21 ± 3.27	32.70 ± 4.01	29.58 ± 3.07	42.50 ± 4.39^#^^∗^	32.27 ± 2.19	49.30 ± 4.37^#^^∗^	33.03 ± 2.79	53.82 ± 8.04^#^^∗^	31.39 ± 3.45	55.53 ± 4.23^#^^∗^	33.37 ± 4.55	65.55 ± 7.52^#^^∗^
LVEF (%)	66.51 ± 10.62	63.57 ± 5.74	66.87 ± 10.64	60.47 ± 7.95	61.93 ± 7.81	50.39 ± 10.96	63.79 ± 7.52	37.13 ± 8.28^#^^∗^	66.94 ± 9.48	32.10 ± 3.07^#^^∗^	67.18 ± 10.85	29.05 ± 7.31^#^^∗^
FS (%)	34.18 ± 4.46	33.82 ± 2.93	32.59 ± 5.04	30.88 ± 7.67	33.44 ± 3.87	29.05 ± 7.74	32.92 ± 3.77	17.70 ± 4.38^#^^∗^	35.18 ± 4.96	16.62 ± 2.94^#^^∗^	30.07 ± 6.28	15.11 ± 4.18^#^^∗^

Values are expressed as mean ± SEM (*n* = 9 mice per group). ^#^Compared with the 0 week, *p* < 0.05. ^∗^Compared with the sham group, *p* < 0.05, one-way ANOVA with the Bonferroni posttest. IVSTd: interventricular septal thickness at diastole; LVPWd: left ventricular posterior wall thickness at diastole; LVIDd: left ventricular internal diameter at end-diastole; LVIDs: left ventricular internal diameter at end-systole; IVRT: isovolumic relaxation time; EDT: E peak deceleration time; EF: left ventricular ejection fraction; FS: fractional shortening.

**Table 3:** Heart rate, blood pressure, and echocardiography parameters in HFpEF mice.

	Sham	HFpEF	HFpEF+low IVA	HFpEF+high IVA
HR (beats per min)	369.25 ± 47.94	483.14 ± 37.49^#^	395.37 ± 19.05^∗^	332.97 ± 33.16^∗^
DBP (mmHg)	80.47 ± 10.71	92.98 ± 4.17^#^	95.77 ± 4.74^#^	95.11 ± 5.06^#^
SBP (mmHg)	128.15 ± 7.59	151.70 ± 8.32^#^	150.42 ± 6.75^#^	153.24 ± 14.10^#^
IVSTd (mm)	0.65 ± 0.04	0.82 ± 0.04^#^	0.78 ± 0.04^#^	0.74 ± 0.05^∗^
LVPWd (mm)	0.66 ± 0.03	0.82 ± 0.06^#^	0.77 ± 0.02^#^	0.70 ± 0.03^∗^
LVIDd (mm)	3.58 ± 0.11	3.85 ± 0.21	3.70 ± 0.29	3.83 ± 0.06
LVIDs (mm)	2.34 ± 0.32	2.42 ± 0.31	2.54 ± 0.37	2.48 ± 0.23
IVRT (ms)	26.60 ± 4.96	42.30 ± 4.57^#^	30.72 ± 9.19	25.48 ± 3.31^∗^
EDT (ms)	32.27 ± 2.19	49.30 ± 4.37^#^	43.07 ± 8.88	37.64 ± 2.07^∗^
LVEF (%)	61.92 ± 7.81	50.39 ± 10.96	52.73 ± 5.17	52.86 ± 6.40
FS (%)	33.44 ± 3.87	29.05 ± 7.74	28.47 ± 6.23	27.46 ± 3.74

Values are expressed as mean ± SEM (*n* = 9 mice per group). ^#^Compared with the sham group, *p* < 0.05. ^∗^Compared with the HFpEF group, *p* < 0.05, one-way ANOVA with the Bonferroni posttest. HR: heart rate; DBP: diastolic blood pressure; SBP: systolic blood pressure; IVSTd: interventricular septal thickness at diastole; LVPWd: left ventricular posterior wall thickness at diastole; LVIDd: left ventricular internal diameter at end-diastole; LVIDs: left ventricular internal diameter at end-systole; IVRT: isovolumic relaxation time; EDT: E peak deceleration time; EF: left ventricular ejection fraction; FS: fractional shortening.

**Table 4:** Heart rate, blood pressure, and echocardiography parameters in HFrEF mice.

	Sham	HFrEF	HFrEF+low IVA	HFrEF+high IVA
HR (beats per min)	393.53 ± 26.27	559.27 ± 52.93^#^	474.47 ± 55.06^#^^∗^	437.74 ± 29.47^∗^
DBP (mmHg)	76.93 ± 7.45	99.22 ± 6.30^#^	105.04 ± 10.87^#^	102.92 ± 8.69^#^
SBP (mmHg)	130.22 ± 7.31	163.63 ± 2.72^#^	160.91 ± 7.70^#^	161.80 ± 4.92^#^
IVSTd (mm)	0.66 ± 0.04	0.95 ± 0.06^#^	0.94 ± 0.04^#^	0.86 ± 0.06^#^
LVPWd (mm)	0.65 ± 0.04	0.97 ± 0.09^#^	0.99 ± 0.07^#^	0.96 ± 0.15^#^
LVIDd (mm)	3.59 ± 0.21	4.22 ± 0.36^#^	4.28 ± 0.05^#^	4.24 ± 0.31^#^
LVIDs (mm)	2.22 ± 0.24	3.44 ± 0.19^#^	3.32 ± 0.11^#^	3.10 ± 0.24^#^
IVRT (ms)	30.37 ± 4.12	58.89 ± 4.19^#^	45.84 ± 9.04^#^	30.84 ± 7.86^∗^
EDT (ms)	33.37 ± 4.55	65.55 ± 7.52^#^	57.78 ± 11.91^#^	40.52 ± 4.71^∗^
LVEF (%)	67.18 ± 10.85	29.05 ± 7.31^#^	45.22 ± 7.16^#^	57.44 ± 9.17^∗^
FS (%)	30.07 ± 6.28	15.11 ± 4.18^#^	21.61 ± 2.93	30.17 ± 7.40^∗^

Values are expressed as mean ± SEM (*n* = 9 mice per group). ^#^Compared with the sham group, *p* < 0.05. ^∗^Compared with the HFrEF group, *p* < 0.05, one-way ANOVA with the Bonferroni posttest. HR: heart rate; DBP: diastolic blood pressure; SBP: systolic blood pressure; IVSTd: interventricular septal thickness at diastole; LVPWd: left ventricular posterior wall thickness at diastole; LVIDd: left ventricular internal diameter at end-diastole; LVIDs: left ventricular internal diameter at end-systole; IVRT: isovolumic relaxation time; EDT: E peak deceleration time; EF: left ventricular ejection fraction; FS: fractional shortening.

## Data Availability

The data used to support the findings of this study are included within the article.

## Supplementary Materials

Supplementary Materials Supplementary Table 1: list of primer pairs used in RT-PCR.
