# Supplementary material for: Ivabradine Ameliorates Cardiac Function in Heart Failure with Preserved and Reduced Ejection Fraction via Upregulation of miR-133a
Source: Oxid Med Cell Longev. 2021 Sep 29;2021:1257283. doi: 10.1155/2021/1257283 (PMC8494584; doi:10.1155/2021/1257283)
Supplement: Supplementary Materials — Supplementary Table 1: list of primer pairs used in RT-PCR. [file 1257283.f1.docx]

**Supplementary Table 1 List of primer pairs used in RT-PCR**

| Gene | Stand | Sequence（5’-3’） |
| --- | --- | --- |
| Mouse CTGF | forward | CCAGTGCAGGGTCCGAGGT |
|  | reverse | GTGCACCATCTTTGGCAGTG |
| Mouse GAPDH | forward | CCAGCTACTCGCGGCTTTA |
|  | reverse | ATCCGTTCACACCGACCTTC |
| Mouse miRNA-133a-3p | forward | TGCGGTTTGGTCCCCTTCAACC |
|  | reverse | AGAACTGTGTACGGAGCGTG |
| Mouse U6 | forward | TGCGGGTGCTCGCTTCGGCAGC |
|  | reverse | CCAGTGCAGGGTCCGAGGT |
| Rat CTGF | forward | CTTCCCGAGAAGGGTCAAGC |
|  | reverse | TTCCAGTCGGTAGGCAGCTA |
| Rat GAPDH | forward | GGCACAGTCAAGGCTGAGAATG |
|  | reverse | ATGGTGGTGAAGACGCCAGTA |
| Rat miRNA-133a-3p | forward | TGCGGTTTGGTCCCCTTCAACC |
|  | reverse | CCAGTGCAGGGTCCGAGGT |
| Rat U6 | forward | TGCGGGTGCCTGCTTCGGCAGC |
|  | reverse | CCAGTGCAGGGTCCGAGGT |
